# Accuracy of Rapid Antigen vs Reverse Transcriptase–Polymerase Chain Reaction Testing for SARS-CoV-2 Infection in College Athletes During Prevalence of the Omicron Variant

**DOI:** 10.1001/jamanetworkopen.2022.17234

**Published:** 2022-06-15

**Authors:** Jessica Tsao, Andrea L. Kussman, Cristina Costales, Benjamin A. Pinsky, Geoffrey D. Abrams, Calvin E. Hwang

**Affiliations:** 1 Department of Orthopaedic Surgery, Stanford University School of Medicine, Stanford, California; 2 Department of Pathology, Stanford University School of Medicine, Stanford, California

## Abstract

This case series evaluates the performance of rapid antigen tests in detecting SARS-CoV-2 infection in college athletes during prevalence of a dominant Omicron variant.

## Introduction

One critical aspect of COVID-19 management has been to isolate or quarantine individuals to prevent viral spread. Reverse transcriptase–polymerase chain reaction (RT-PCR) tests are the criterion standard for diagnosis of SARS-CoV-2 infection but are limited by long turnaround times and complex laboratory methods.

Rapid antigen tests (RATs) can be performed and interpreted in minutes without the use of special equipment. With the rise of each new variant, the accuracy of RATs must be reevaluated. This case series evaluated the performance of RATs in detecting SARS-CoV-2 in the context of a dominant Omicron variant.

## Methods

All Stanford University student athletes who reported no history of SARS-CoV-2 infection in the previous 90 days and returned to campus from January 1 through January 11, 2022, self-administered an RAT (BinaxNOW; Abbott Laboratories) within 24 hours of campus arrival. Symptomatic students with a positive RAT result were isolated without confirmatory RT-PCR testing; asymptomatic students with a positive RAT result underwent RT-PCR testing (eMethods in the Supplement).

Statistical analysis was performed using Prism, version 9.0 (GraphPad Software), for data visualization and Mann-Whitney *U* tests for median cycle threshold (Ct) value comparison, with 2-sided *P* < .05 indicating statistical significance. This study was approved with a waiver of informed consent by the Stanford University Institutional Review Board. The reporting guideline for case series was used for this study.

## Results

Participants included 723 students aged 17 to 23 years (376 [52.0%] female; 709 [98%] received 2-dose Pfizer or Moderna or 1-dose Johnson & Johnson vaccines). Forty-six participants (6.4%) had positive RAT findings, of whom 35 (76.1%) had symptomatic infections. Among these 35 participants, 12 received a positive RT-PCR result within 24 hours, whereas the remaining 23 were presumed to have positive results (Table). Twenty-seven participants had a negative RAT result followed by a positive RT-PCR result within 24 hours, for a total of 73 diagnosed SARS-CoV-2 infections in the included cohort (infectivity rate, 73 of 723 [10.1%]). Overall, RAT had a sensitivity of 63.0% (95% CI, 51.9%-74.1% [46 of 73]) and specificity of 99.8% (95% CI, 99.5%-100% [1 of 650]). Among symptomatic participants, RAT had a sensitivity of 77.8% (95% CI, 65.6%-89.9% [35 of 45]); among asymptomatic patients, 39.2% (95% CI, 21.2%-57.4% [11 of 28]).

**Table.** RAT vs RT-PCR Testing

RAT result	RT-PCR–positive findings		RT-PCR–negative findings
	Confirmed	Presumed	
Positive, No. of participants	23	23	1
Negative, No. of participants	27	0	649
Sensitivity, % (95% CI) [No./total No.]	63.0 (51.9-74.1) [46/73]		NA
Specificity, % (95% CI) [No./total No.]	NA		99.8 (99.5-100) [649/650]

Abbreviations: NA, not applicable; RAT, rapid antigen testing; RT-PCR, reverse transcriptase–polymerase chain reaction.

Participants with RAT- and RT-PCR–positive findings (n = 23) had a lower median Ct value of 24.6 (IQR, 22.2-32.3) compared with those with RAT-negative and RT-PCR–positive findings (n = 27), with a median Ct value of 35.0 (IQR, 29.8-36.6; *P* < .001) (Figure). In the RT-PCR–positive cohort, symptomatic individuals (n = 22) had lower Ct values compared with their asymptomatic counterparts (24.7 [IQR, 22.4-31.9] vs 33.6 [IQR, 29.3-35.7]; *P* = .004).

**Figure.**
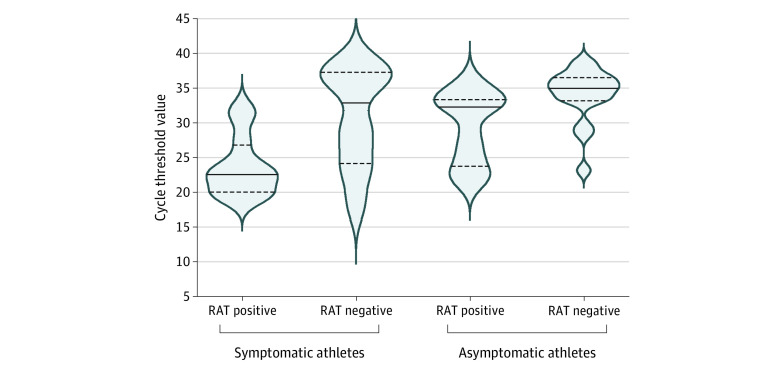
SARS-CoV-2 Reverse Transcriptase–Polymerase Chain Reaction Cycle Threshold Values by Rapid Antigen Test (RAT) Results and COVID-19 Symptoms Data are from 723 student athletes undergoing testing for SARS-CoV-2. Solid lines indicate median cycle threshold value; dotted lines, cycle threshold value quartiles.

In the RT-PCR analysis, the Omicron variant represented 44 of 46 positive cases (95.7%). Four specimens could not be genotyped owing to low viral loads.

## Discussion

The overall infectivity rate in our cohort was 10.1%, with 95.7% of infections due to the Omicron variant. The overall sensitivity of RAT was 63.0%, similar to that of 2 recent studies^1,2^ that also focused on the Omicron variant. Performance of RAT was better in symptomatic individuals, likely owing to the significant difference in Ct values, because Ct values have been shown to correlate to the sensitivity of RAT in both laboratory^3,4^ and clinical studies^1^. Rapid antigen tests were quite specific, with only 1 false-positive finding in the cohort, suggesting that a positive RAT finding in both asymptomatic and symptomatic populations does not require confirmatory RAT or RT-PCR testing. This is consistent with previous studies that have shown excellent specificity.^5,6^

Study limitations include self-administration and self-reporting of RAT findings, which could confound our results; however, RATs are frequently administered in this fashion. In addition, not every symptomatic patient with a positive RAT finding underwent confirmatory RT-PCR testing, which could cause elevated specificity values.

Rapid antigen testing performed similarly in the detection of the Omicron variant compared with previous variants, with high specificity but poor sensitivity, particularly among asymptomatic individuals. Its use as a screening tool for asymptomatic infection in the setting of widespread prevalence of the Omicron variant may be limited.

## Notes

Supplement. **eMethods.** Reverse Transcriptase–Polymerase Chain Reaction Testing
eReferences
