# Supplementary material for: Accuracy of Rapid Antigen vs Reverse Transcriptase–Polymerase Chain Reaction Testing for SARS-CoV-2 Infection in College Athletes During Prevalence of the Omicron Variant
Source: JAMA Netw Open. 2022 Jun 15;5(6):e2217234. doi: 10.1001/jamanetworkopen.2022.17234 (PMC9201670; doi:10.1001/jamanetworkopen.2022.17234)
Supplement: Supplement. — eMethods. Reverse Transcriptase–Polymerase Chain Reaction Testing eReferences [file jamanetwopen-e2217234-s001.pdf]

Supplementary Online Content

Tsao J, Kussman AL, Costales C, Pinsky BA, Abrams GD, Hwang CE. Accuracy of rapid antigen vs reverse transcriptase–polymerase chain reaction testing for SARS-CoV-2 infection in college athletes during prevalence of the Omicron variant. *JAMA Netw Open*. 2022;5(6):e2217234. doi:10.1001/jamanetworkopen.2022.17234

eMethods. Reverse Transcriptase–Polymerase Chain Reaction Testing
eReferences

This supplementary material has been provided by the authors to give readers additional information about their work.

© 2022 Tsao J et al. *JAMA Network Open*.

eMethods. Reverse Transcriptase–Polymerase Chain Reaction Testing

RT-PCRs were performed by the Stanford Clinical Virology Laboratory using one of two comparable platforms: the Panther Fusion SARS-CoV-2 Assay (Hologic Inc., San Diego, CA, USA) or PerkinElmer SARS-CoV-2 Assay (Waltham, MA, USA) with cycle threshold (Ct) values recorded for the Orf1ab targets. Specimens underwent an RT-PCR for spike insertion-deletion_211-214 specific for the Omicron variant (B.1.1.529, BA.1). Samples negative for this Omicron-specific mutation were genotyped as previously described.[1]

eReferences

1. Wang H, Miller JA, Verghese M, et al. Multiplex SARS-CoV-2 genotyping reverse transcriptase PCR for population-level variant screening and epidemiologic surveillance. J Clin Microbiol. 2021;59(8):e0085921. doi:10.1128/JCM.00859-21

© 2022 Tsao J et al. *JAMA Network Open*.
